# *Ex Situ* Conservation Priorities for the Wild Relatives of Potato (*Solanum* L. Section *Petota*)

**DOI:** 10.1371/journal.pone.0122599

**Published:** 2015-04-29

**Authors:** Nora P. Castañeda-Álvarez, Stef de Haan, Henry Juárez, Colin K. Khoury, Harold A. Achicanoy, Chrystian C. Sosa, Vivian Bernau, Alberto Salas, Bettina Heider, Reinhard Simon, Nigel Maxted, David M. Spooner

**Affiliations:** 1 Decision and Policy Analysis Program, International Center for Tropical Agriculture (CIAT), Cali, Colombia; 2 School of Biosciences, University of Birmingham, Birmingham, United Kingdom; 3 Global Program Genetic Resources, International Potato Center (CIP), Lima, Peru; 4 Centre for Crop Systems Analysis, Wageningen University, Wageningen, The Netherlands; 5 Integrated IT and Computational Research Unit, International Potato Center (CIP), Lima, Peru; 6 USDA-ARS, Vegetable Crop Research Unit, Department of Horticulture, University of Wisconsin, Madison, Wisconsin, United States of America

## Abstract

Crop wild relatives have a long history of use in potato breeding, particularly for pest and disease resistance, and are expected to be increasingly used in the search for tolerance to biotic and abiotic stresses. Their current and future use in crop improvement depends on their availability in *ex situ* germplasm collections. As these plants are impacted in the wild by habitat destruction and climate change, actions to ensure their conservation *ex situ* become ever more urgent. We analyzed the state of *ex situ* conservation of 73 of the closest wild relatives of potato (*Solanum* section *Petota*) with the aim of establishing priorities for further collecting to fill important gaps in germplasm collections. A total of 32 species (43.8%), were assigned high priority for further collecting due to severe gaps in their *ex situ* collections. Such gaps are most pronounced in the geographic center of diversity of the wild relatives in Peru. A total of 20 and 18 species were assessed as medium and low priority for further collecting, respectively, with only three species determined to be sufficiently represented currently. Priorities for further collecting include: (i) species completely lacking representation in germplasm collections; (ii) other high priority taxa, with geographic emphasis on the center of species diversity; (iii) medium priority species. Such collecting efforts combined with further emphasis on improving *ex situ* conservation technologies and methods, performing genotypic and phenotypic characterization of wild relative diversity, monitoring wild populations *in situ*, and making conserved wild relatives and their associated data accessible to the global research community, represent key steps in ensuring the long-term availability of the wild genetic resources of this important crop.

## Introduction

Potato (*Solanum tuberosum* L.) is the most important tuber crop worldwide, continuing to gain significance in temperate and tropical regions as a source of carbohydrates, vitamins, and minerals [1] as well as for industrial purposes [2]. The crop is susceptible to a wide range of biotic stresses, in particular fungal diseases and pests [3,4]. A relatively low historical influx of variation has led to a genetic bottleneck within potato cultivars [5–7], thus the development of potato varieties with novel genetic diversity is expected to improve resistance to biotic and abiotic constraints [8].

As one source of such variation, potato breeding programs have looked to related wild species [8–10]. Widely used and well documented sources of valuable traits such as frost and late blight (*Phytophthora infestans* (Mont.) de Bary) resistance include *S*. *acaule*, *S*. *bulbocastanum*, *S*. *chacoense*, *S*. *demissum* and *S*. *stoloniferum*. The search for late blight resistance *has been a center point in the evaluation and use of wild relatives in potato breeding [11–15]. In addition*, S. commersonii *and S*. *berthaultii* have been evaluated for bacterial wilt (*Ralstonia solanacearum* Smith) and verticillium wilt (*Verticillium* spp.) resistances, respectively [16–18]. Other species have been proposed as valuable sources of resistance, e.g., *S*. *acroglossum* for Colorado potato beetle (*Leptinotarsa decemlineata* Say), and *S*. *albicans* for cold sweetening [19,20] (Table 1).

**Table 1:** Crop wild relatives that have been evaluated and/or used in potato breeding.

Genepool	Species	Resistance trait(s)	Reference
Primary	*S*. *acaule*	Biotic: *Nacobbus aberrans*. Abiotic: frost	[21–24]
	*S*. *berthaultii*	Biotic: *Erwinia carotovora*, *E*. *atroseptica*; *Verticillium* wilt. Other: cold induced sweetening	[25–28]
	*S*. *brevicaule*	Biotic: *Globodera* sp., *G*. *pallida*, virus	[22,29–31]
	*S*. *candolleanum*	Biotic: *Globodera* sp., *G*. *pallida*, *Erwinia carotovora*, *E*. *atroseptica*	[25,26,31]
	*S*. *vernei*	Biotic: virus, pest and nematode	[22,31,32]
Secondary	*S*. *boliviense*	Abiotic: frost	[33,34]
	*S*. *cajamarquense*	Biotic: *Phytophthora infestans*	[35]
	*S*. *chacoense*	Biotic: virus, pest, *Verticillium* wilt Other: cold induced sweetening	[27,28,30,36,37]
	*S*. *demissum*	Biotic: *Phytophthora infestans*	[36,38]
	*S*. *kurtzianum*	Biotic: *Globodera* sp.	[31]
	*S*. *paucissectum*	Biotic: *Phytophthora infestans*	[39]
	*S*. *raphanifolium*	Other: cold induced sweetening	[28]
	*S*. *stoloniferum*	Biotic: Phytophthora infestans, PVY	[32,36]
Tertiary	*S*. *bulbocastanum*	Biotic: *Phytophthora infestans*	[12,40,41]
	*S*. *commersonii*	Biotic: *Ralstonia solanacearum*. Abiotic: frost	[16,18,42]
	*S*. *palustre*	Biotic: PLRV	[43]
	*S*. *tarnii*	Biotic: PVY, *Leptinotarsa decemlineata*, *Phytophthora infestans*	[44]

Despite the extensive history of use of the wild relatives of potato in breeding, most species have not yet been evaluated for their potential for utilization. These include species from the eastern Andean slopes where resistance to late blight is particularly key for survival (e.g. *S*. *laxissimum* and *S*. *rhomboideilanceolatum*), as well as more distant relatives that may display drought resistance due to their adaptation to dry habitats (e.g. *S*. *immite* and *S*. *mochiquense*). Enhanced understanding of species reproductive biologies, advances in pre-breeding technologies to bypass reproductive barriers, improvements in cisgenic techniques, and the evolution of new genotyping and phenotyping platforms are likely make the use of wild relatives more attractive and efficient [45–49].

**Fig 1:**
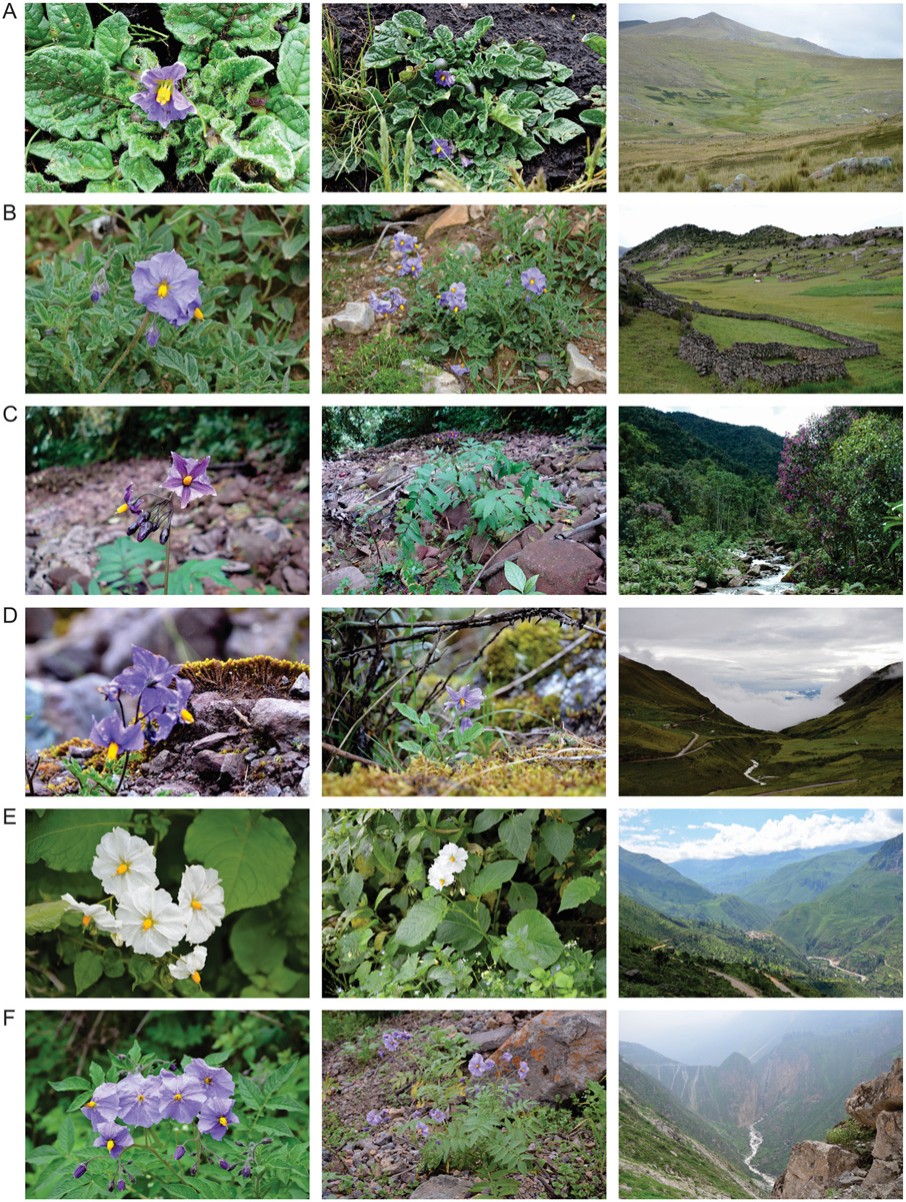
Flowers, plants and habitats of six potato wild relatives. A) *Solanum acaule*, B) *S*. *candolleanum*, C) *S*. *laxissimum*, D) *S*. *rhomboideilanceolatum*, E) *S*. *simplicissimum* and F) *S*. *wittmackii*. Photographs by S. de Haan. The author of the photographs has given written consent to publish them.

Species designations within the section *Petota*, where potato resides, have recently been revised on the basis of new molecular findings in combination with morphological studies [50–55]. The wild related species of potato have been organized into primary, secondary and tertiary genepools according to the ease of crossability with the cultivated species [56,57]. These wild relatives constitute a morphologically and genetically diverse group of plants distributed from central Chile and Argentina to the southwestern United States. They occupy a variety of habitats within deserts, forests and mountainous regions [58] (Fig 1). Mexico, Bolivia, Argentina, and especially Peru are considered to possess the greatest total diversity of potato wild relatives, although high levels of endemism are reflected in unique species occurring in most of the total 16 countries where these wild relatives grow [58].

While CWR are likely to play a role in climate change adaptation of novel potato cultivars [59], a number of the wild relatives of cultivated potato are threatened due to habitat destruction and climate change [60–62]. It is therefore becoming more important to address gaps in the *ex situ* conservation of these plants, particularly for species that are currently underrepresented in genebanks and are most impacted in their native habitats.

Gap analysis is a systematic methodology for assessing the comprehensiveness of *ex situ* conservation of plant species, and for assigning taxonomic and geographic priorities for further collecting [63,64]. Gap analysis has been applied to the wild relatives of a wide range of crops, including grains, forages and legumes [57,64,65]. The analysis can also contribute to the identification of species and habitat priorities for complementary *in situ* conservation.

Here we assessed the current state of *ex situ* conservation of the wild relatives of potato through a gap analysis, in order to identify those species and geographic areas in need of conservation in order to assure their long-term availability for plant breeding efforts.

## Materials and Methods

### Wild relative species and geographic area of study

We assessed the closely related wild relatives of potato (i.e. primary and secondary genepool wild relatives [66]), as well as any distant relatives in the third genepool that have been reported with confirmed or potential uses in crop breeding (Table 2). We followed the most recent taxonomic revision of *Solanum* L. section *Petota* [55] (see also Solanaceae Source, http://solanaceaesource.org/), henceforth “Solanaceae Source taxonomy”. A complementary analysis was also performed following the taxonomy of Ochoa [67–69] (henceforth “CIP taxonomy”), in order to provide a gap analysis for the potato wild relative collection conserved as the International Potato Center (CIP), based on its current taxonomic classification (S1 Table). Our study focused on the native distributions of potato wild relatives, which occur in Argentina, Bolivia, Brazil, Chile, Colombia, Costa Rica, Ecuador, Guatemala, Honduras, Mexico, Panama, Paraguay, Peru, Uruguay, USA, and Venezuela [55].

**Table 2:** List of 73 species analyzed and their corresponding prioritization category, genepool, ploidy level, native areas and count of data retrieved for this study.

Species scientific name	Countries	Ploidy [70] and (EBN)[71]	Genepool	No. of reference samples (georeferenced)	No. of germplasm accessions (georeferenced)	SRS	GRS	ERS	FPS	FPCAT
*S*. *acaule* Bitter	ARG; BOL; PER; CHL	4x (2EBN), 6x	Primary	3058 (864)	1762 (521)	3.66	10.00	10.00	7.89	NFCR
*S*. *acroglossum* Juz.	PER	2x (2EBN)	Secondary	92 (23)	4 (4)	0.42	0.61	3.00	0.00	HPS
*S*. *acroscopicum* Ochoa	PER	2x	Secondary	93 (38)	11 (7)	1.06	0.90	6.36	2.77	HPS
*S*. *agrimonifolium* Rydberg	GTM; HND; MEX	4x (2EBN)	Secondary	345 (118)	40 (14)	1.04	6.48	4.21	3.91	MPS
*S*. *albicans* (Ochoa) Ochoa	ECU; PER	6x (4EBN)	Secondary	288 (73)	132 (40)	3.14	5.20	10.00	6.11	LPS
*S*. *albornozii* Correll	ECU	2x (2EBN)	Secondary	25 (7)	13 (8)	3.42	5.06	7.50	5.33	LPS
*S*. *andreanum* Baker	COL; ECU	2x (2EBN); 4x (4EBN)	Secondary	448 (234)	111 (71)	1.99	5.06	6.47	4.51	MPS
*S*. *ayacuchense* Ochoa	PER	2x (2EBN)	Secondary	10 (7)	0 (0)	0.00	0.00	0.00	0.00	HPS
*S*. *berthaultii* J. G. Hawkes	ARG; BOL	2x (2EBN), 3x	Primary	836 (292)	323 (116)	2.79	7.68	10.00	6.82	LPS
*S*. *boliviense* M. F. Dunal in DC.	BOL; PER; ARG	2x (2EBN)	Secondary	1724 (657)	388 (185)	1.84	8.00	10.00	6.61	LPS
*S*. *bombycinum* C. M. Ochoa	BOL	4x	Secondary	8 (6)	1 (1)	1.11	1.62	5.00	0.00	HPS
*S*. *brevicaule* Bitter	ARG; BOL; PER	2x (2EBN); 4x (4EBN); 6x (4EBN)	Primary	4428 (1477)	1159 (457)	2.07	10.00	10.00	7.36	LPS
*S*. *buesii* Vargas	PER	2x (2EBN)	Secondary	63 (32)	6 (4)	0.87	0.24	2.73	0.00	HPS
*S*. *bulbocastanum* Dunal in Poiret	GTM; MEX	2x (1EBN), 3x	Tertiary	970 (399)	175 (47)	1.53	6.20	10.00	5.91	LPS
*S*. *burkartii* Ochoa	PER	2x	Secondary	88 (18)	7 (5)	0.74	6.09	8.33	0.00	HPS
*S*. *cajamarquense* Ochoa	PER	2x (1EBN)	Secondary	223 (39)	16 (8)	0.67	1.06	6.00	2.58	HPS
*S*. *candolleanum* Berthault	PER; BOL	2x (2EBN), 3x	Primary	2910 (1245)	739 (349)	2.03	10.00	9.17	7.06	LPS
*S*. *cantense* Ochoa	PER	2x (2EBN)	Secondary	155 (68)	3 (3)	0.19	0.93	3.75	0.00	HPS
*S*. *chacoense* Bitter	ARG; BOL; PRY; PER; URY; BRA	2x (2EBN), 3x	Secondary	2527 (1004)	710 (119)	2.19	1.94	5.52	3.22	MPS
*S*. *chilliasense* Ochoa	ECU	2x (2EBN)	Secondary	15 (7)	5 (4)	2.50	10.00	10.00	0.00	HPS
*S*. *chiquidenum* Ochoa	PER	2x (2EBN)	Secondary	360 (148)	17 (11)	0.45	3.27	7.00	3.57	MPS
*S*. *chomatophilum* Bitter	PER; ECU	2x (2EBN)	Secondary	967 (378)	124 (55)	1.14	6.54	8.33	5.34	LPS
*S*. *clarum* D. S. Correll	GTM; MEX	2x	Secondary	244 (92)	6 (4)	0.24	3.69	2.78	0.00	HPS
*S*. *colombianum* Dunal	COL; ECU; PAN; VEN	4x (2EBN)	Secondary	1116 (444)	214 (105)	1.61	6.47	9.14	5.74	LPS
*S*. *commersonii* M. F. Dunal	ARG; BRA; URY	2x (1EBN), 3x	Tertiary	692 (272)	112 (30)	1.39	2.14	5.83	3.12	MPS
*S*. *contumazaense* Ochoa	PER	2x (2EBN)	Secondary	21 (13)	2 (2)	0.87	5.26	6.67	0.00	HPS
*S*. *demissum* Lindley	GTM; MEX	6x (4EBN)	Secondary	1669 (513)	613 (85)	2.69	8.30	10.00	6.99	LPS
*S*. *flahaultii* Bitter	COL	4x	Secondary	99 (37)	39 (10)	2.83	2.66	3.75	3.08	MPS
*S*. *gandarillasii* H. M. Cárdenas	BOL	2x (2EBN)	Secondary	48 (28)	21 (7)	3.04	3.72	7.14	4.64	MPS
*S*. *garcia-barrigae* Ochoa	COL	4x	Secondary	21 (10)	3 (2)	1.25	0.52	1.90	0.00	HPS
*S*. *gracilifrons* Bitter	PER	2x	Secondary	19 (8)	1 (1)	0.50	1.47	3.75	0.00	HPS
*S*. *guerreroense* D. S. Correll	MEX	6x (4EBN)	Secondary	4 (2)	20 (2)	8.33	10.00	10.00	9.44	NFCR
*S*. *hastiforme* Correll	PER	2x (2EBN)	Secondary	49 (32)	2 (2)	0.39	0.38	4.00	0.00	HPS
*S*. *hintonii* D. S. Correll	MEX	2x	Secondary	39 (18)	0 (0)	0.00	0.00	0.00	0.00	HPS
*S*. *hjertingii* J. G. Hawkes	MEX	4x (2EBN)	Secondary	155 (62)	54 (10)	2.58	1.93	4.00	2.84	HPS
*S*. *hougasii* D. S. Correll	MEX	6x (4EBN)	Secondary	186 (79)	39 (10)	1.73	2.12	3.68	2.51	HPS
*S*. *huancabambense* Ochoa	PER	2x (2EBN)	Secondary	111 (28)	29 (10)	2.07	2.07	5.56	3.23	MPS
*S*. *incasicum* Ochoa	PER	2x (2EBN)	Secondary	9 (5)	2 (2)	1.82	10.00	5.00	0.00	HPS
*S*. *infundibuliforme* R. A. Philippi	ARG; BOL	2x (2EBN)	Primary	836 (277)	234 (116)	2.19	4.71	7.78	4.89	MPS
*S*. *iopetalum* (Bitter) J. G. Hawkes	MEX	6x (4EBN)	Secondary	626 (313)	93 (51)	1.29	5.23	7.50	4.67	MPS
*S*. *kurtzianum* Bitter & L. Wittmack	ARG	2x (2EBN)	Secondary	764 (253)	276 (32)	2.65	4.02	8.75	5.14	LPS
*S*. *laxissimum* Bitter	PER	2x (2EBN)	Secondary	139 (91)	19 (10)	1.20	1.73	5.00	2.64	HPS
*S*. *lesteri* J. G. Hawkes & Hjerting	MEX	2x	Secondary	23 (12)	12 (4)	3.43	4.22	4.44	4.03	MPS
*S*. *limbaniense* Ochoa	PER	2x (2EBN)	Secondary	56 (28)	12 (7)	1.76	1.18	5.00	2.65	HPS
*S*. *lobbianum* Bitter	COL	4x (2EBN)	Secondary	1 (1)	4 (1)	8.00	NA	NA	0.00	HPS
*S*. *longiconicum* Bitter	CRI; PAN	4x	Secondary	546 (198)	25 (12)	0.44	10.00	10.00	6.81	LPS
*S*. *maglia* D. F. L. von Schlechtendal	CHL; ARG	2x, 3x	Secondary	190 (51)	15 (4)	0.73	0.14	1.33	0.74	HPS
*S*. *medians* Bitter	PER; CHL	2x (2EBN), 3x	Secondary	849 (305)	98 (35)	1.03	4.32	4.44	3.27	MPS
*S*. *microdontum* Bitter	ARG; BOL	2x (2EBN), 3x	Secondary	1178 (349)	422 (94)	2.64	6.25	9.09	5.99	LPS
*S*. *morelliforme* Bitter & Muench	GTM; MEX; HND	2x	Secondary	364 (140)	45 (18)	1.10	4.74	6.55	4.13	MPS
*S*. *multiinterruptum* Bitter	PER	2x (2EBN), 3x	Secondary	496 (204)	95 (45)	1.61	7.33	8.75	5.90	LPS
*S*. *neocardenasii* J. G. Hawkes & J. P. Hjerting	BOL	2x	Secondary	25 (17)	17 (5)	4.05	0.56	3.64	2.75	HPS
*S*. *neorossii* J. G. Hawkes & J. P. Hjerting	ARG	2x	Secondary	76 (35)	45 (14)	3.72	4.17	10.00	5.96	LPS
*S*. *neovavilovii* Ochoa	BOL	2x (2EBN)	Secondary	26 (13)	0 (0)	0.00	0.00	0.00	0.00	HPS
*S*. *nubicola* Ochoa	PER	4x (2EBN)	Secondary	36 (20)	2 (2)	0.53	0.70	5.45	0.00	HPS
*S*. *okadae* J. G. Hawkes & J. P. Hjerting	BOL	2x	Primary	139 (55)	75 (19)	3.50	1.08	7.14	3.91	MPS
*S*. *olmosense* Ochoa	ECU; PER	2x (2EBN)	Secondary	26 (15)	0 (0)	0.00	0.00	0.00	0.00	HPS
*S*. *oxycarpum* Schiede in D. F. L. von Schlechtendal	MEX	4x (2EBN)	Secondary	203 (77)	58 (20)	2.22	2.45	7.93	4.20	MPS
*S*. *paucissectum* Ochoa	PER	2x (2EBN)	Secondary	182 (20)	20 (10)	0.99	10.00	10.00	7.00	LPS
*S*. *pillahuatense* Vargas	PER	2x (2EBN)	Secondary	15 (11)	1 (1)	0.63	10.00	10.00	0.00	HPS
*S*. *piurae* Bitter	PER	2x (2EBN)	Secondary	226 (38)	17 (7)	0.70	0.47	3.00	1.39	HPS
*S*. *polyadenium* Greenman	MEX	2x	Secondary	286 (97)	99 (14)	2.57	3.52	8.13	4.74	MPS
*S*. *raphanifolium* Cárdenas & Hawkes	PER	2x (2EBN)	Secondary	597 (206)	220 (69)	2.69	6.52	8.57	5.93	LPS
*S*. *rhomboideilanceolatum* Ochoa	PER	2x (2EBN)	Secondary	99 (46)	7 (3)	0.66	1.04	6.67	0.00	HPS
*S*. *salasianum* Ochoa	PER	2x	Secondary	13 (7)	0 (0)	0.00	0.00	0.00	0.00	HPS
*S*. *schenckii* Bitter	MEX	6x (4EBN)	Secondary	105 (37)	49 (13)	3.18	2.45	6.80	4.14	MPS
*S*. *sogarandinum* Ochoa	PER	2x (2EBN), 3x	Secondary	157 (81)	27 (13)	1.47	3.22	6.67	3.79	MPS
*S*. *stoloniferum* D. F. L. von Schlechtendal	MEX; USA	4x (2EBN)	Secondary	3807 (1464)	1582 (314)	2.94	10.00	10.00	7.65	NFCR
*S*. *tarnii* J. G. Hawkes & Hjerting	MEX	2x	Tertiary	68 (31)	45 (10)	3.98	2.58	4.62	3.73	MPS
*S*. *venturii* J. G. Hawkes & J. P. Hjerting	ARG	2x (2EBN)	Secondary	165 (62)	39 (6)	1.91	0.47	4.44	2.28	HPS
*S*. *vernei* Bitter & L. Wittmack	ARG	2x (2EBN)	Primary	429 (122)	261 (47)	3.78	2.46	8.89	5.04	LPS
*S*. *verrucosum* D. F. L. von Schlechtendal	MEX	2x (2EBN), 3x, 4x	Secondary	968 (378)	222 (36)	1.87	6.56	5.91	4.78	MPS
*S*. *violaceimarmoratum* Bitter	BOL; PER	2x (2EBN)	Secondary	234 (104)	61 (16)	2.07	0.98	2.86	1.97	HPS

SRS: Sampling Representativeness Score., GRS: Geographical Representativeness Score., ERS: Environmental Representativeness Score., FPS: Final priority score., FPCAT: Final priority category., HPS = high priority species, MPS = medium priority species, LPS = low priority species, and NFCR = ‘no further collecting required’ (NFCR). ARG: Argentina, BOL: Bolivia, BRA: Brazil, CHL: Chile, COL: Colombia, CRI: Costa Rica, ECU: Ecuador, GTM: Guatemala, HND: Honduras, MEX: Mexico, PAN: Panama, PER: Peru, PRY: Paraguay, URY: Uruguay, USA: United States of America and VEN: Venezuela.

Germplasm data were obtained from repositories that provide straightforward access to genetic resources and associated data to the global research community through online information systems (i.e. EURISCO -http://eurisco.ipk-gatersleben.de/, GRIN -http://www.ars-grin.gov/- and CIP’s biomart portal -http://germplasmdb.cip.cgiar.org/-). Species presence records and additional germplasm accessions passport data were gathered from online databases and via communications with data managers (i.e. GBIF -http://www.gbif.org/-, CRIA -http://splink.cria.org.br/-, SINGER, CPNWH -http://www.pnwherbaria.org/-, the Atlas of Guatemalan Crop Wild Relatives [72], “PBI Solanum—a worldwide treatment”, “LAC biosafety”, CAS, F, FSU, H and MANCH)), extracted from the literature [55], and through visits to herbaria (i.e. E, K, L, NY, MA, PH, RB and US). The occurrence data utilized in this analysis is available on http://dx.doi.org/10.6084/m9.figshare.1284187.

### Environmental niche modelling

Environmental niche modelling (ENM) techniques were used to estimate the potential geographic distribution of each wild potato species. MaxEnt [73] was selected as the modelling algorithm due to its performance when compared with other modelling approaches, and to its wide use in conservation analyses [74–76]. Ten thousand random points were used as background records across Central and South America, the native range of the wild relatives. A five-fold cross-validation option (k = 5) was implemented to maximize the use of small sets of georeferenced records in the modelling, producing five replicates per species, subsequently summarized into a single ensemble model by estimating the mean values across the replicates. The models were restricted to their known native countries per species as reported in the literature [55], and further refined using a species-specific threshold corresponding to the shortest distance to the upper left corner of the Receiver Operating Characteristic (ROC) curve [77]. For environmental drivers, we used 19 bioclimatic variables (S2 Table) derived from the WorldClim database [78] at a resolution of 2.5 arc-minutes (approx. 5 km at the equator).

The performance of each ENM was assessed to determine its suitability for use in the gap analysis. Three parameters were checked: (i) the 5-fold average Area Under the Test ROC Curve (ATAUC), (ii) the standard deviation of the ATAUC for the 5 different folds, and (iii) the proportion of potential distribution where the standard deviation is greater than 0.15 (ASD15). A suitable model had to meet these conditions: ATAUC >0.7, STAUC <0.15 and ASD15 <10% [64]. In those cases where a suitable niche model was not produced (either due to lack of data or low performance of the ensemble model), a convex hull (polygon surrounding the outermost georeferenced points) was prepared.

### Gap analysis

We used a gap analysis methodology [63,64] including three metrics to determine the urgency of collecting wild relatives for conservation *ex situ*. A Sampling Representativeness Score (SRS) compared the number of germplasm accessions to the total number of samples (germplasm plus species presence records, with or without geographic coordinates), giving a general overview of the sufficiency of accessions per species. A Geographic Representativeness Score (GRS) compared the ENMs of the species to the geographic distribution of existing germplasm accession collecting sites, estimated by creating circular buffers of 50 km (CA50) around each site where the accession was collected [79], in order to assess the geographic coverage of germplasm collections. An Ecosystem Representativeness Score (ERS) assessed the number of ecosystems currently represented in *ex situ* collections (CA50 of germplasm collections), in comparison to the total number of ecosystems distributed within the ENMs of species. For this, a world terrestrial ecoregions map was used to determine the ecosystem units [80]. The three gap analysis metrics were given equal weight and an average was calculated to obtain a Final Priority Score (FPS). Four categories were employed to assign priority for further collecting for *ex situ* conservation: high priority species (HPS) when FPS ≤3, or when ten or less accessions were recorded in germplasm collections; medium-priority species (MPS) when 3< FPS ≥5; low priority species (LPS) when 5< FPS ≥7.5; and ‘no further collecting of germplasm required’ (NFCR) when 7.5< FPS ≥10.

The gap analysis was performed using R v2.15.1 [81], and the packages maptools [82], rgdal [83], SDMTools [84], raster [85], sp [86,87], dismo [88] and ggplot2 [89].

### Identification of geographic areas of priority for further collecting

Maps highlighting areas identified as priorities for further collecting (collecting gaps) were prepared for each species by subtracting the existing germplasm CA50 buffers from the ENMs. For those species where a niche model was not produced, CA50 buffers were prepared around all presence records, with germplasm CA50 buffers subtracted from these representations of the distribution of species. Collecting gap maps for all high priority species were analyzed using the “Zonal Statistics” tool in ArcMap 10.1 to produce a count of species in need of further collecting per country.

## Results

### Wild relative species and geographic area of study

**Fig 2:**
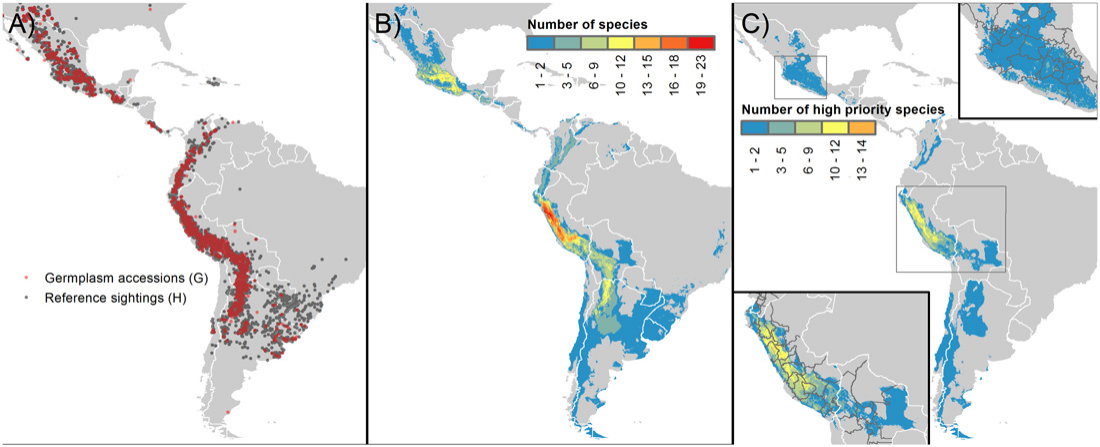
Distribution of the wild relatives of potato and hotspots for collecting. A) Distribution of germplasm and herbarium records included in the analysis. Red dots represent germplasm accessions (G) and dark gray dots herbarium/presence records (H). B) Species richness based upon environmental niche models, and C) Potential hotspots for further collecting of high priority species (HPS).

Seventy-three species were included in the analysis as relatively close relatives of potato (i.e. members of the primary and secondary genepools [66] or due to published actual or potential use in breeding efforts). These included seven species from the primary genepool of potato, 63 from the secondary genepool, and three tertiary genepool species with reported use in crop improvement (Table 2). Almost half of the species analyzed are diploids with an endosperm balance number of 2 (2 EBN), followed by tetraploids (2 EBN and 4 EBN) and hexaploids (4 EBN) [71]. For the complementary gap analysis, following the CIP taxonomy, a total of 187 putative species were analyzed, equivalent to the 73 Solanaceae Source taxonomy species [55] (S1 Table). A total of 49,164 records for the 73 potato wild relatives were gathered (75.76% with coordinates), with 11,100 germplasm accessions and 37,251 presence records, including herbarium references, inactive germplasm accessions, and field sighting recordings (Fig 2A).

### Environmental niche modelling

The environmental niche models of 75 species (89%) met the parameters used to consider an ENM suitable for use in the gap analysis. For the remaining eight species (*S*. *chilliasense*, *S*. *guerreroense*, *S*. *incasicum*, *S*. *lobbianum*, *S*. *neovavilovii*, *S*. *olmosense*, *S*. *paucissectum*, and *S*. *pillahuatense*), convex hulls were prepared and used in the gap analysis, as the ENM replicates produced were highly variable and did not comply with the ASD15 condition. Potato crop wild relative species richness was found to be highest in Peru, followed by Mexico and Argentina (Fig 2B, S1 File).

Occurrence data, ENMs and the collecting priorities maps for the species analyzed, following the Solanaceae Source taxonomy, are available in an interactive format at http://www.cwrdiversity.org/distribution-map/.

### Gap analysis

**Fig 3:**
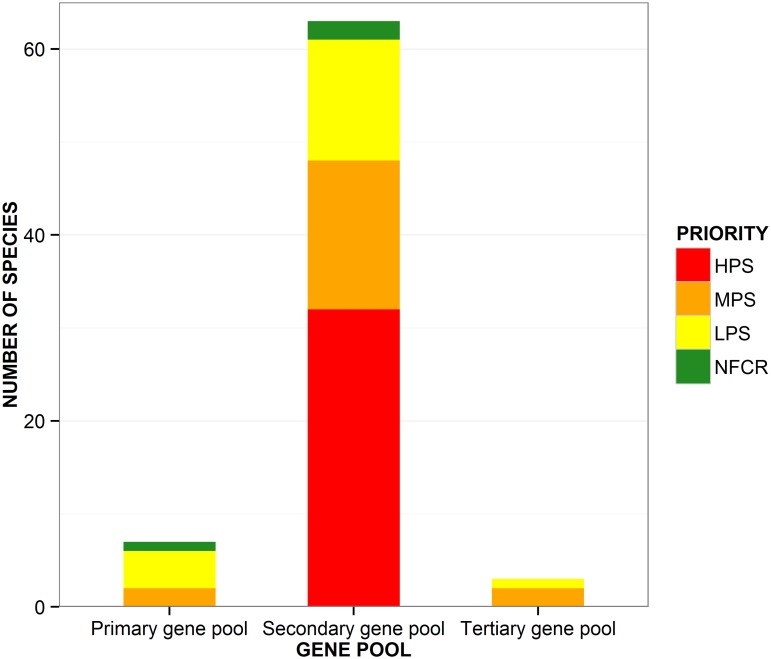
Potato wild relatives’ priorities for further collecting by genepool. Categories are: high priority species (HPS), medium priority species (LPS), low priority species (LPS), and ‘no further collecting required’ (NFCR).

The gap analysis for the 73 species resulted in the assignment of 32 HPS, 20 MPS, 18 LPS and 3 NFCR (Table 2). There are no germplasm accessions currently available for *S*. *ayacuchense*, *S*. *neovavilovii*, *S*. *olmosense* and *S*. *salasianum*, and these species therefore represent the greatest urgency for further collecting. All HPS belong to the secondary genepool (Fig 3).

**Fig 4:**
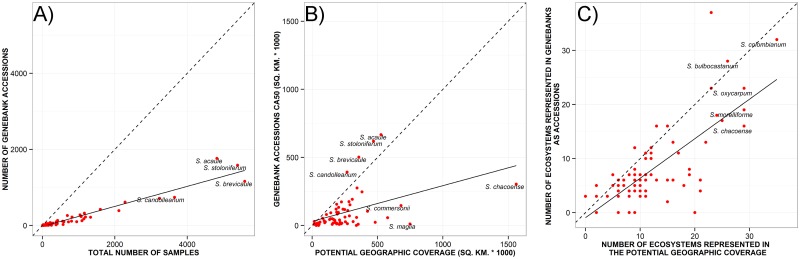
Gap analysis metrics. A) Sampling Representativeness Score (SRS), B) Geographic Representativeness Score (GRS), and C) Ecosystem Representativeness Score (ERS) gap analysis metrics for potato wild relatives. Red dots represent results per species. Dashed lines represent complete representativeness in *ex situ* conservation systems. A linear regression (continuous lines) depicts the mean trend for the genepool.

*Solanum neocardenasii* and *S*. *lobbianum* possessed a single dominant factor contributing to their priority category assignment for further collecting. All other species possessed two (40.6% of the species), three (28.1%) or four (28.1%) factors contributing importantly to their FPS status (S3 Table). Ninety-four percent of the species classified as HPS had a low SRS (SRS equal or less than 3) [median (mean) = 0.73 (1.22)] (Fig 4A, S1 Fig). Likewise, 78.1% of HPS exhibited a low GRS [0.930 (2.07)] (S1 Fig), with five species well represented (*S*. *candolleanum*, *S*. *brevicaule*, *S*. *stoloniferum* and *S*. *acaule*), as shown in Fig 4B, where the dashed line is the complete representativeness line, and the continuous line is the average representativeness line, the former showing an ideal scenario where the potential geographic extension of the genepool is completely represented at genebank collections and the latter showing the extent of representativeness compared to the potential extent of the genepool. On the other hand, the ERS contributed less to the FPS of high priority species, with less than half (37.5%) of the HPS exhibiting an ERS ≤3 [median value 3.75 (4.01)] (Fig 4, S1 Fig). A total of 65.6% of the species ranked as high priority had less than ten active accessions and consequently very limited representativeness in terms of absolute numbers of accessions available in germplasm collections.

**Fig 5:**
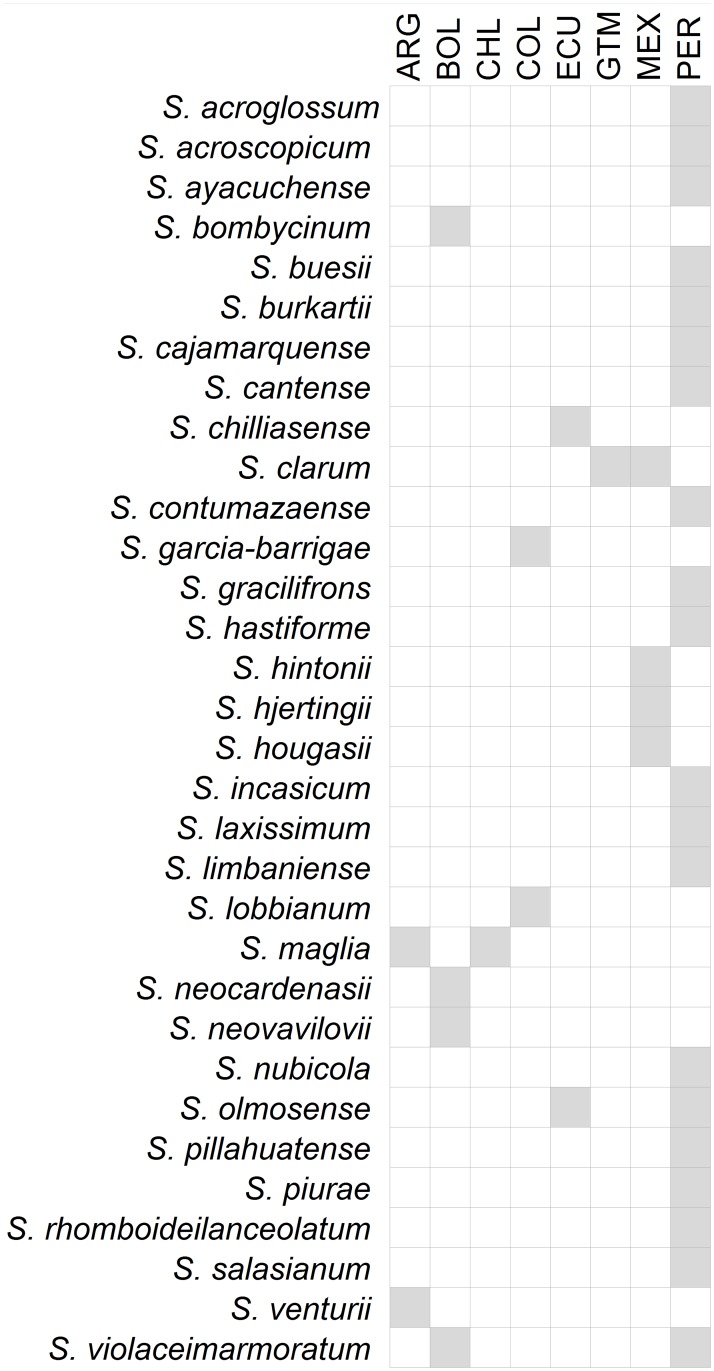
Countries identified for potential further collecting per high priority crop wild relative species. ARG: Argentina, BOL: Bolivia, CHL: Chile, COL: Colombia, ECU: Ecuador, GTM: Guatemala, MEX: Mexico, PER: Peru.

A total of 31 HPS were mapped together for targeting of geographic hotspots for further collecting (Fig 2C, S2 File). Peru contained the highest count of HPS for further collecting (21 species), followed by Mexico (4); Bolivia (3); Colombia (2), Ecuador (2) and Argentina, Chile and Guatemala (each with 1 species) (Fig 2C). Twenty-eight species (out of 32) were found to be endemic to a single country (Fig 5). The greatest concentrations of species requiring further collecting were predicted to occur in the Peruvian Departments of Cajamarca, La Libertad, Ancash and Huánuco. S4 Table provides an overview of sites recommended for further collecting of high priority species based on their presence points.

**Fig 6:**
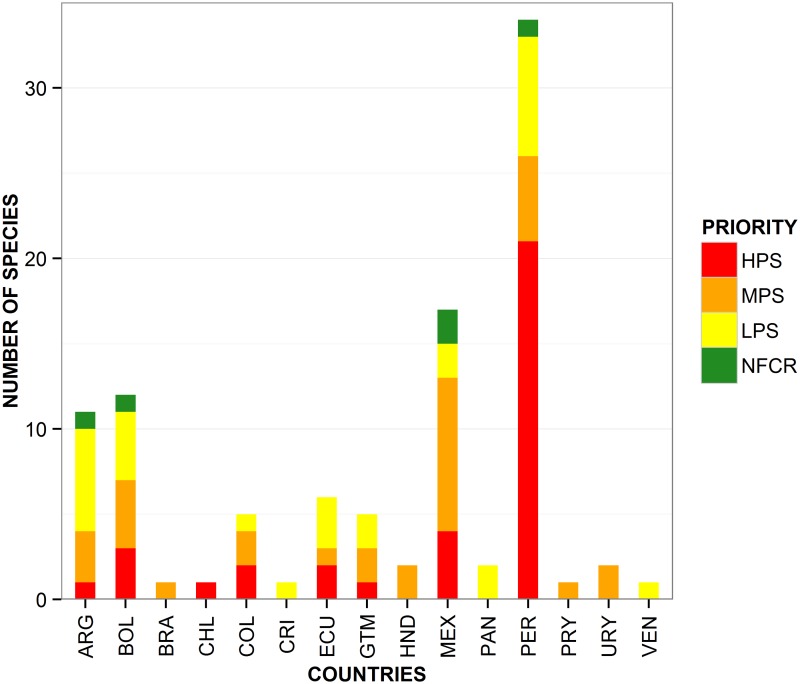
Number of CWR species prioritized for further collecting per country. HPS = high priority species, MPS = medium priority species, LPS = low priority species, and NFCR = ‘no further collecting required’ (NFCR). ARG: Argentina, BOL: Bolivia, BRA: Brazil, CHL: Chile, COL: Colombia, CRI: Costa Rica, ECU: Ecuador, GTM: Guatemala, HND: Honduras, MEX: Mexico, PAN: Panama, PER: Peru, PRY: Paraguay, URY: Uruguay and VEN: Venezuela.

A total of 18 species were assessed as MPS for further collecting, and are distributed in: Argentina (1 species), Bolivia (2), Colombia (1), Ecuador (2), Guatemala (2), Mexico (8), Peru (5), Honduras (2), Paraguay (1), Uruguay (1) and Brazil (1) (Fig 6).

The restricted range and endemic nature of many of the insufficiently collected taxa implies that targeted collecting trips to specific regions outside the gap richness areas are needed in order to form comprehensive germplasm collections for potato wild relatives. Some of the HPS species are known to occur in threatened habits, requiring urgent attention; e.g. *S*. *rhomboideilanceolatum* (Fig 1D) and *S*. *piurae*. Other species, such as *S*. *laxissimum* (Fig 1C) and *S*. *neovavilovii*, occur in relatively intact natural areas or within the boundaries of national parks and can thus be expected to be more secure. Active monitoring of these species in the wild can provide greater assurance of continued conservation in these areas.

## Discussion

With 32 species classified as high priority and another 20 as medium priority for collecting, it is evident that further conservation action is needed to safeguard the wild genetic resources of this globally important crop. We propose three levels of priority for further collecting: first for the four HPS species that are completely lacking from internationally available genebank collections (*S*. *ayacuchense*, *S*. *neovavilovii*, *S*. *olmosense* and *S*. *salasianum*); second for the other 28 HPS species occurring in a total of eight countries; and third for the MPS.

In addition to gap filling for *ex situ* collections, the results can help establish priorities for the establishment of genetic reserves for the *in situ* conservation of potato wild relatives. Such reserves may most effectively be established at sites where several HPS and/or MPS overlap, especially if coinciding with existing protected areas. Habitats undergoing significant disturbance may also represent high priorities for consideration for *in situ* conservation efforts.

Some of the HPS display very restricted distributions and are considered to be threatened *in situ*. The limited habitat of *S*. *rhomboideilanceolatum* in Peru is increasingly exposed to road building and overgrazing by livestock (field observation by the authors, 2013). Yet other HPS with restricted distributions, such as *S*. *bombycinum* in Bolivia, are reported to grow in habitats that are not presently highly exposed to threats [62], while additional species with relatively extensive ranges such as *S*. *laxissimum* in Peru show considerable spatial overlap with protected areas. Factors such as threats to the *in situ* conservation of wild populations, overlap with protected areas, and degree of endemism can further refine collecting priorities. Monitoring the population dynamics, ecology and genetics of selected species to corroborate the effect of climate change and other threats to wild relatives also represent useful contributions to conservation planning [90]. Such studies can help to ground-truth climate change forecasts and to enhance the understanding of the adaptive capacity of wild relatives.

Many of the taxa classified as generally well conserved (LPS and NFCR) are those that are widely used in breeding programs, such as *S*. *bulbocastanum* and *S*. *stoloniferum*. This is a logical consequence of demand from such programs. It is anticipated that demand for as yet underutilized species will increase as potato breeding efforts expand the use of wide diversity in order to confront emerging biotic and abiotic stresses.

Our results assign a relatively large number of species from Peru to the category of high priority for further collecting. This may seem surprising given the long history of collecting missions in the center of species diversity. Sampling biases relative to road systems, time limitations of collecting missions and the tendency of collectors to sample in areas of previous expeditions have been reported [58,91]. The high levels of endemism, and difficult access to some of the areas where HPS potato wild relatives occur provide further insight into the low level of representation of a number of these species in genebanks. New roads in Peru in previously isolated and remote habitats will soon make these populations increasingly accessible for collecting but at the same time more vulnerable to habitat destruction.

Long-term conservation of the genetic diversity of wild relatives of potato will also require further research in population genetics and reproductive biology of the species [92]. Gap filling of the taxa identified here as critically under-represented in germplasm collections will provide an important step in making germplasm available for such analyses. Future studies should incorporate morphological and molecular analyses in order to elucidate the diversity and genetic distances within and between populations of wild relatives as well as between genebank collections and *in situ* reserves [93–95]. Genetic variability encountered within natural populations of CWR has been described in few cases [96,97] but has not generally been taken into account when planning collecting expeditions for wild relatives [98]. Further taxonomic research may also be useful. The complementary gap analysis following the CIP taxonomy displayed differences in resulting priorities for further collecting (S2 Fig), and may reveal potentially useful infraspecific variation for further exploration, as some of the species in CIP taxonomy may represent unique subpopulations within the Solanaceae Source taxonomy.

The collecting priorities identified here, combined with further emphasis on improving *ex situ* conservation technologies and associated data management, performing genotypic and phenotypic characterization of wild relative diversity, monitoring wild populations *in situ*, and making conserved wild relatives and their associated data accessible to the global research community, represent key steps in ensuring the long-term availability of the wild genetic resources of this critically important crop.

## Supporting Information

S1 Fig Boxplots showing the values obtained for the Gap Analysis metrics. Sampling Representativeness Score (SRS), Geographic Representativeness Score (GRS) and Ecosystem Representativeness Score (ERS), ordered by high priority species (HPS), medium priority species (MPS), low priority species (LPS), and ‘no further collecting required’ (NFCR).
(TIFF)

S2 Fig Share of species per prioritization category by taxonomic classification system. High priority species (HPS), medium priority species (LPS), low priority species (LPS), and ‘no further collecting required’ (NFCR).
(TIFF)

S1 File Species richness map for further exploration in Google Earth. (ZIP)

S2 File Potential hotspots for further collecting of high priority species (HPS) for further exploration in Google Earth. (ZIP)

S1 Table List of 172 species following CIP taxonomy, its equivalences in Solanaceae Source Taxonomy [55] and the prioritization category obtained through the gap analysis. SRS: Sampling Representativeness Score, GRS: Geographical Representativeness Score, ERS: Environmental Representativeness Score, FPCAT: Final priority category.
(DOCX)

S2 Table List of bioclimatic variables [99] used as environmental drivers to produce environmental niche models. C.V.: coefficient of variation
(DOCX)

S3 Table High priority species for further collecting and the main factors contributing to insufficient representation in germplasm collections. (DOCX)

S4 Table List of regions and localities where further collecting may be targeted per species. (DOCX)
