# Correction: *Ex Situ* Conservation Priorities for the Wild Relatives of Potato (*Solanum* L. Section *Petota*)

**DOI:** 10.1371/journal.pone.0129873

**Published:** 2015-06-03

**Authors:** The PLOS ONE Staff

The Academic Editor field does not appear on this article. The Academic Editor is John P. Hart, New York State Museum, United States. The publisher apologizes for the error.
