# Supplementary figures and images for: Ex Situ Conservation Priorities for the Wild Relatives of Potato (Solanum L. Section Petota)
Source: PLoS One. 2015 Apr 29;10(4):e0122599. doi: 10.1371/journal.pone.0122599 (PMC4414521; doi:10.1371/journal.pone.0122599)

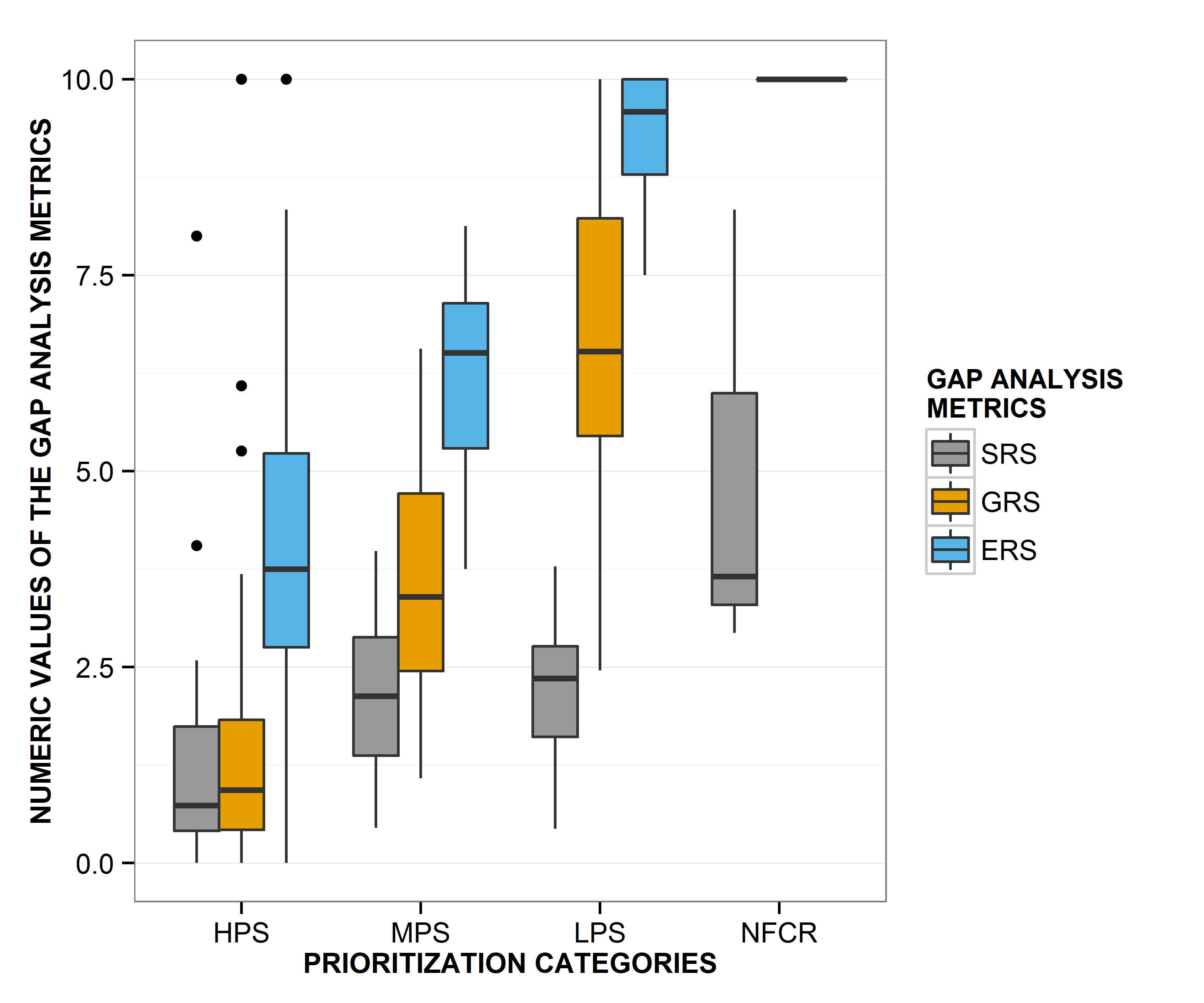

Supplement: S1 Fig — Sampling Representativeness Score (SRS), Geographic Representativeness Score (GRS) and Ecosystem Representativeness Score (ERS), ordered by high priority species (HPS), medium priority species (MPS), low priority species (LPS), and ‘no further collecting required’ (NFCR). (TIFF) [file pone.0122599.s001.tiff]

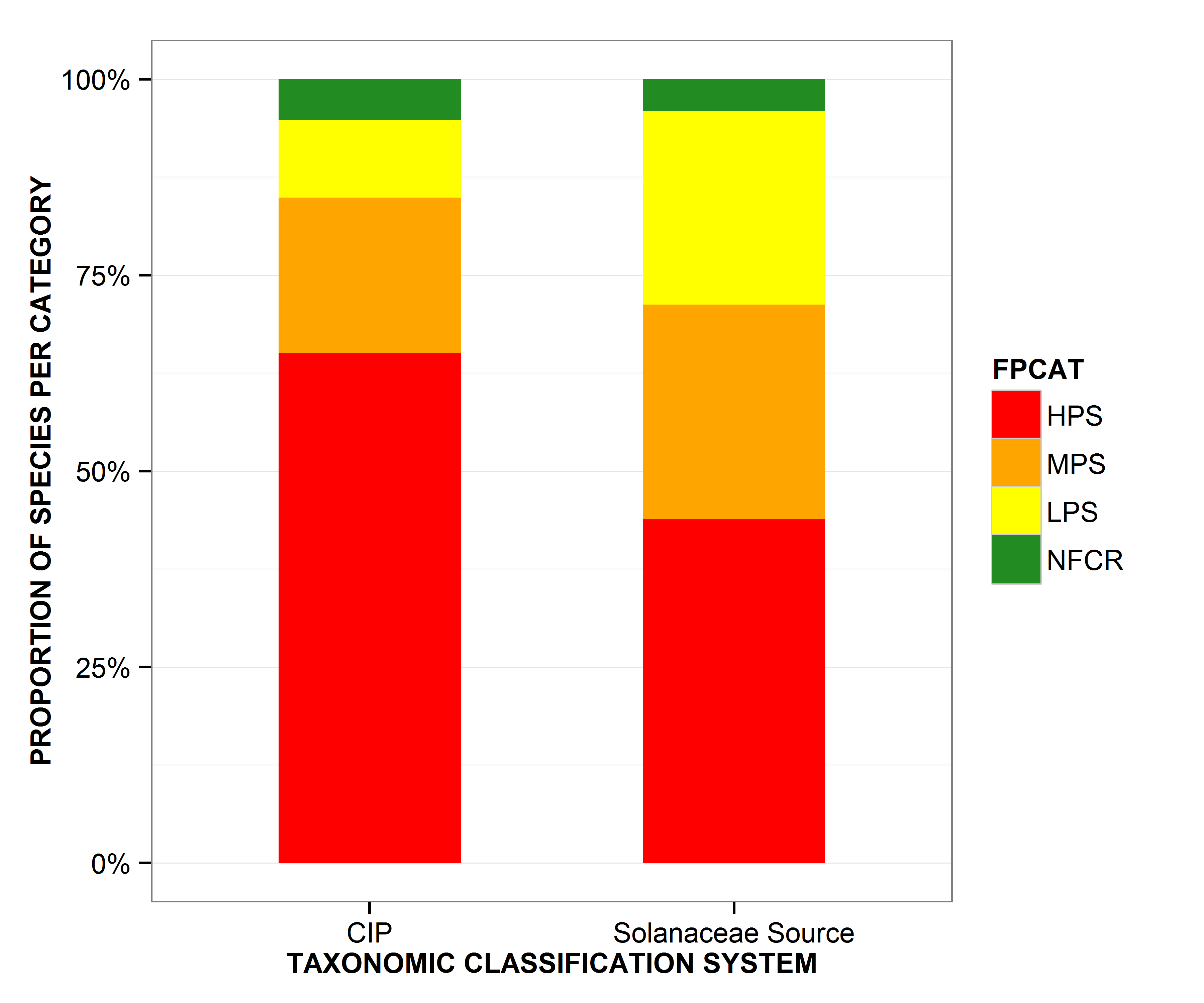

Supplement: S2 Fig — High priority species (HPS), medium priority species (LPS), low priority species (LPS), and ‘no further collecting required’ (NFCR). (TIFF) [file pone.0122599.s002.tiff]
