# Supplementary material for: Ex Situ Conservation Priorities for the Wild Relatives of Potato (Solanum L. Section Petota)
Source: PLoS One. 2015 Apr 29;10(4):e0122599. doi: 10.1371/journal.pone.0122599 (PMC4414521; doi:10.1371/journal.pone.0122599)
Supplement: S1 Table — SRS: Sampling Representativeness Score, GRS: Geographical Representativeness Score, ERS: Environmental Representativeness Score, FPCAT: Final priority category. (DOCX) [file pone.0122599.s005.docx]

**S1 Table. List of 172 species following CIP taxonomy, its equivalences in Solanaceae Source Taxonomy [55] and the prioritization category obtained through the gap analysis.** SRS: Sampling Representativeness Score, GRS: Geographical Representativeness Score, ERS: Environmental Representativeness Score, FPCAT: Final priority category.

| **Species name (CIP taxonomy)** | **Species name (equivalent in Solanaceae source taxonomy [55])** | **No. of records** | **No. of records with coordinates** | **SRS** | **GRS** | **ERS** | **FPS** | **FPCAT** |
| --- | --- | --- | --- | --- | --- | --- | --- | --- |
| *S. acaule* | *S. acaule* | 4820 | 1349 | 3.655602 | 10 | 10 | 7.885201 | NFCR |
| *S. achacachense* | *S. candolleanum* | 2 | 2 | 10 | 10 | 10 | 0 | HPS |
| *S. acroglossum* | *S. acroglossum* | 96 | 26 | 0.416667 | 0.612331 | 2.727273 | 0 | HPS |
| *S. acroscopicum* | *S. acroscopicum* | 28 | 18 | 1.071429 | 1.500815 | 5 | 0 | HPS |
| *S. agrimonifolium* | *S. agrimonifolium* | 385 | 130 | 1.038961 | 6.475075 | 4 | 3.838012 | MPS |
| *S. alandiae* | *S. brevicaule* | 22 | 14 | 8.636364 | 10 | 10 | 9.545455 | NFCR |
| *S. albicans* | *S. albicans* | 420 | 111 | 3.142857 | 5.195343 | 9.230769 | 5.856323 | LPS |
| *S. albornozii* | *S. albornozii* | 38 | 13 | 3.421053 | 5.05852 | 6 | 4.826524 | MPS |
| *S. amayanum* | *S. candolleanum* | 9 | 7 | 3.333333 | 10 | 6 | 0 | HPS |
| *S. ambosinum* | *S. candolleanum* | 48 | 31 | 3.125 | 3.244488 | 6 | 4.123163 | MPS |
| *S. anamatophilum* |  | 4 | 4 | 0 | 0 | 0 | 0 | HPS |
| *S. ancophilum* | *S. rhomboideilanceolatum* | 18 | 11 | 3.333333 | 4.10788 | 7.142857 | 0 | HPS |
| *S. ancoripae* | *S. candolleanum* | 5 | 4 | 2 | 10 | 10 | 0 | HPS |
| *S. andreanum* | *S. andreanum* | 37 | 20 | 5.405405 | 10 | 10 | 8.468468 | NFCR |
| *S. ariduphilum* | *S. chiquidenum* | 4 | 3 | 0 | 0 | 0 | 0 | HPS |
| *S. augustii* |  | 10 | 9 | 3 | 10 | 5 | 0 | HPS |
| *S. avilesii* | *S. brevicaule* | 7 | 3 | 5.714286 | 10 | 10 | 0 | HPS |
| *S. ayacuchense* | *S. ayacuchense* | 10 | 7 | 0 | 0 | 0 | 0 | HPS |
| *S. aymaraesense* | *S. candolleanum* | 4 | 3 | 0 | NA | NA | 0 | HPS |
| *S. berthaultii* | *S. berthaultii* | 39 | 22 | 8.205128 | 3.155475 | 5.555556 | 5.638719 | LPS |
| *S. bill-hookeri* | *S. candolleanum* | 7 | 6 | 0 | 0 | 0 | 0 | HPS |
| *S. boliviense* | *S. boliviense* | 17 | 12 | 7.647059 | 1.567983 | 5.714286 | 4.976442 | MPS |
| *S. bombycinum* | *S. bombycinum* | 9 | 7 | 1.111111 | 1.620354 | 4.285714 | 0 | HPS |
| *S. brevicaule* | *S. brevicaule* | 24 | 12 | 7.5 | 0.999647 | 5 | 4.499882 | MPS |
| *S. buesii* | *S. buesii* | 69 | 34 | 0.869565 | 0.242759 | 2.5 | 0 | HPS |
| *S. bukasovii* | *S. candolleanum* | 973 | 441 | 3.56629 | 10 | 5.454545 | 6.340278 | LPS |
| *S. bulbocastanum* | *S. bulbocastanum* | 1145 | 421 | 1.528384 | 6.059595 | 10 | 5.86266 | LPS |
| *S. burkartii* | *S. burkartii* | 2 | 2 | 0 | NA | NA | 0 | HPS |
| *S. burtonii* | *S. andreanum* | 3 | 2 | 0 | NA | NA | 0 | HPS |
| *S. cajamarquense* | *S. cajamarquense* | 239 | 45 | 0.669456 | 1.064599 | 5.454545 | 2.3962 | HPS |
| *S. calacalinum* | *S. colombianum* | 3 | 2 | 6.666667 | 10 | 10 | 0 | HPS |
| *S. calvescens* | *S. chacoense* | 2 | 2 | 0 | NA | NA | 0 | HPS |
| *S. candolleanum* | *S. candolleanum* | 34 | 21 | 5.882353 | 1.82812 | 4.117647 | 3.942707 | MPS |
| *S. cantense* | *S. cantense* | 158 | 71 | 0.189873 | 0.930109 | 3.333333 | 0 | HPS |
| *S. cardiophyllum* |  | 6 | 2 | 5 | 10 | 10 | 0 | HPS |
| *S. chacoense* | *S. chacoense* | 94 | 6 | 2.659574 | 0.172606 | 2.5 | 1.777394 | HPS |
| *S. chancayense* |  | 15 | 9 | 2 | 1.655976 | 3.333333 | 0 | HPS |
| *S. chilliasense* | *S. chilliasense* | 20 | 10 | 2.5 | 10 | 10 | 0 | HPS |
| *S. chillonamum* | *S. candolleanum* | 6 | 5 | 5 | 2.957084 | 6 | 0 | HPS |
| *S. chiquidenum* | *S. chiquidenum* | 207 | 73 | 0.628019 | 3.626488 | 5.454545 | 3.236351 | MPS |
| *S. chomatophilum* | *S. chomatophilum* | 406 | 153 | 1.847291 | 6.176781 | 6.923077 | 4.982383 | MPS |
| *S. circaeifolium* |  | 127 | 1 | 0 | NA | NA | 0 | HPS |
| *S. clarum* | *S. clarum* | 250 | 93 | 0.24 | 3.687675 | 2.631579 | 0 | HPS |
| *S. coelestispetalum* | *S. candolleanum* | 97 | 52 | 3.71134 | 1.775876 | 3.636364 | 3.041193 | MPS |
| *S. colombianum* | *S. colombianum* | 140 | 40 | 3.785714 | 2.991093 | 4.864865 | 3.880557 | MPS |
| *S. commersonii* | *S. commersonii* | 804 | 296 | 1.393035 | 2.037383 | 5.384615 | 2.938344 | HPS |
| *S. contumazaense* | *S. contumazaense* | 23 | 14 | 0.869565 | 5.256465 | 5.714286 | 0 | HPS |
| *S. demissum* | *S. demissum* | 2282 | 544 | 2.68624 | 7.546396 | 9.2 | 6.477545 | LPS |
| *S. dolichocremastrum* |  | 57 | 32 | 3.157895 | 5.866814 | 5 | 4.674903 | MPS |
| *S. donachui* | *S. garcia-barrigae* |  |  |  |  |  |  | HPS |
| *S. ehrenbergii* |  | 4 | 2 | 7.5 | 10 | 10 | 0 | HPS |
| *S. flahaultii* | *S. flahaultii* | 16 | 5 | 9.375 | 10 | 10 | 9.791667 | NFCR |
| *S. flavoviridens* | *S. berthaultii* | 2 | 2 | 0 | NA | NA | 0 | HPS |
| *S. gandarillasii* | *S. gandarillasii* | 69 | 34 | 3.043478 | 3.724284 | 6.25 | 4.339254 | MPS |
| *S. garcia-barrigae* | *S. garcia-barrigae* | 2 | 1 | 5 | NA | NA | 0 | HPS |
| *S. gracilifrons* | *S. gracilifrons* | 20 | 9 | 0.5 | 1.469502 | 3.333333 | 0 | HPS |
| *S. guerreroense* | *S. guerreroense* | 24 | 3 | 8.333333 | 10 | 10 | 9.444444 | NFCR |
| *S. guzmanguense* |  | 6 | 2 | 1.666667 | 10 | 10 | 0 | HPS |
| *S. hastiforme* | *S. hastiforme* | 51 | 33 | 0.392157 | 0.378815 | 3.636364 | 0 | HPS |
| *S. hintonii* | *S. hintonii* | 39 | 18 | 0 | 0 | 0 | 0 | HPS |
| *S. hjertingii* | *S. hjertingii* | 209 | 67 | 2.583732 | 1.925262 | 3.75 | 2.752998 | HPS |
| *S. hoopesii* | *S. brevicaule* | 2 | 2 | 10 | NA | 10 | 0 | HPS |
| *S. hougasii* | *S. hougasii* | 225 | 82 | 1.733333 | 2.118489 | 3.5 | 2.450608 | HPS |
| *S. huancabambense* | *S. huancabambense* | 140 | 36 | 2.071429 | 2.067287 | 5 | 3.046238 | MPS |
| *S. huancavelicae* | *S. candolleanum* | 5 | 3 | 0 | 0 | 0 | 0 | HPS |
| *S. huarochiriense* | *S. chomatophilum* | 58 | 26 | 2.068966 | 3.060428 | 4.285714 | 3.138369 | MPS |
| *S. humectophilum* |  | 378 | 42 | 0.079365 | 0.230647 | 3.076923 | 0 | HPS |
| *S. hygrothermicum* |  | 1 | 1 | 0 | NA | NA | 0 | HPS |
| *S. hypacrarthrum* |  | 47 | 30 | 1.06383 | 2.165809 | 2.5 | 0 | HPS |
| *S. immite* |  | 29 | 21 | 2.068966 | 7.341672 | 5.714286 | 0 | HPS |
| *S. incahuasinum* |  | 3 | 2 | 3.333333 | 10 | 10 | 0 | HPS |
| *S. incamayoense* | *S. brevicaule* | 5 | 3 | 10 | 10 | 10 | 0 | HPS |
| *S. incasicum* | *S. incasicum* | 11 | 7 | 1.818182 | 10 | 5 | 0 | HPS |
| *S. infundibuliforme* | *S. infundibuliforme* | 1070 | 380 | 2.186916 | 4.706341 | 7 | 4.631085 | MPS |
| *S. ingifolium* |  | 4 | 4 | 2.5 | 10 | 10 | 0 | HPS |
| *S. iopetalum* | *S. iopetalum* | 719 | 330 | 1.293463 | 5.256712 | 6.923077 | 4.491084 | MPS |
| *S. irosinum* | *S. burkartii* | 77 | 13 | 0.649351 | 10 | 7.142857 | 0 | HPS |
| *S. jaenense* | *S. colombianum* | 3 | 3 | 0 | NA | NA | 0 | HPS |
| *S. jalcae* | *S. chomatophilum* | 50 | 22 | 1.2 | 3.161071 | 5 | 0 | HPS |
| *S. jamesii* |  | 38 | 18 | 10 | 2.275392 | 3.529412 | 5.268268 | LPS |
| *S. kurtzianum* | *S. kurtzianum* | 1040 | 282 | 2.653846 | 4.01721 | 7.777778 | 4.816278 | MPS |
| *S. laxissimum* | *S. laxissimum* | 42 | 31 | 1.904762 | 1.403808 | 3.846154 | 0 | HPS |
| *S. leptophyes* | *S. brevicaule* | 93 | 66 | 5.053763 | 3.082162 | 8.125 | 5.420308 | LPS |
| *S. lesteri* | *S. lesteri* | 35 | 15 | 3.428571 | 4.221037 | 4 | 3.883203 | MPS |
| *S. lignicaule* |  | 23 | 14 | 3.913043 | 10 | 10 | 0 | HPS |
| *S. limbaniense* | *S. limbaniense* | 68 | 33 | 1.764706 | 1.181236 | 4.444444 | 2.463462 | HPS |
| *S. lobbianum* | *S. lobbianum* | 5 | 1 | 8 | NA | NA | 0 | HPS |
| *S. longiconicum* | *S. longiconicum* | 571 | 207 | 0.437828 | 10 | 10 | 6.812609 | LPS |
| *S. longiusculus* | *S. candolleanum* | 4 | 3 | 0 | 0 | 0 | 0 | HPS |
| *S. lopez-camarenae* | *S. acroscopicum* | 9 | 6 | 1.111111 | 8.049098 | 5.714286 | 0 | HPS |
| *S. maglia* | *S. maglia* | 205 | 52 | 0.731707 | 0.309143 | 1.666667 | 0.902506 | HPS |
| *S. marinasense* | *S. candolleanum* | 207 | 77 | 2.173913 | 3.334883 | 5 | 3.502932 | MPS |
| *S. medians* | *S. medians* | 174 | 104 | 2.873563 | 3.459573 | 5.714286 | 4.015807 | MPS |
| *S. megistacrolobum* | *S. boliviense* | 90 | 56 | 4.888889 | 3.758885 | 6.153846 | 4.933873 | MPS |
| *S. microdontum* | *S. microdontum* | 1600 | 425 | 2.6375 | 6.253033 | 8.333333 | 5.741289 | LPS |
| *S. minutifoliolum* |  | 10 | 6 | 2 | 10 | 6.666667 | 0 | HPS |
| *S. mochiquense* |  | 28 | 17 | 2.5 | 8.09517 | 7.142857 | 0 | HPS |
| *S. morelliforme* | *S. morelliforme* | 409 | 147 | 1.100244 | 4.740626 | 6.333333 | 4.058068 | MPS |
| *S. moscopanum* | *S. colombianum* | 14 | 3 | 7.142857 | 10 | 10 | 0 | HPS |
| *S. multiinterruptum* | *S. multiinterruptum* | 591 | 244 | 1.607445 | 7.333468 | 7.777778 | 5.572897 | LPS |
| *S. nemorosum* | *S. colombianum* | 2 | 2 | 0 | NA | NA | 0 | HPS |
| *S. neocardenasii* | *S. neocardenasii* | 42 | 20 | 4.047619 | 0.559267 | 3.333333 | 2.64674 | HPS |
| *S. neorossii* | *S. neorossii* | 118 | 45 | 3.559322 | 3.036364 | 7.5 | 4.698562 | MPS |
| *S. neovalenzuelae* | *S. flahaultii* | 2 | 1 | 10 | NA | NA | 0 | HPS |
| *S. neovargasii* | *S. laxissimum* | 1 | 1 | 0 | NA | NA | 0 | HPS |
| *S. neovavilovii* | *S. neovavilovii* | 26 | 13 | 0 | 0 | 0 | 0 | HPS |
| *S. nubicola* | *S. nubicola* | 38 | 22 | 0.526316 | 0.695551 | 5 | 0 | HPS |
| *S. okadae* | *S. okadae* | 214 | 68 | 3.504673 | 2.090892 | 6.666667 | 4.087411 | MPS |
| *S. olmosense* | *S. olmosense* | 26 | 15 | 0 | 0 | 0 | 0 | HPS |
| *S. oplocense* | *S. brevicaule* | 135 | 68 | 7.555556 | 5.539527 | 9 | 7.365027 | LPS |
| *S. orocense* | *S. colombianum* | 1 | 1 | 10 | NA | NA | 0 | HPS |
| *S. orophilum* | *S. candolleanum* | 68 | 49 | 2.647059 | 8.246238 | 6.25 | 5.714432 | LPS |
| *S. ortegae* | *S. candolleanum* | 1 | 1 | 0 | NA | NA | 0 | HPS |
| *S. otites* | *S. colombianum* | 5 | 3 | 8 | 10 | 5 | 0 | HPS |
| *S. oxycarpum* | *S. oxycarpum* | 261 | 89 | 2.222222 | 2.450221 | 7.666667 | 4.113036 | MPS |
| *S. pampasense* | *S. candolleanum* | 11 | 8 | 7.272727 | 3.641538 | 5 | 0 | HPS |
| *S. pamplonense* | *S. colombianum* | 1 | 1 | 0 | NA | NA | 0 | HPS |
| *S. pascoense* | *S. chomatophilum* | 2 | 2 | 10 | 10 | 10 | 0 | HPS |
| *S. paucijugum* | *S. andreanum* | 26 | 16 | 6.923077 | 4.829122 | 8.571429 | 6.774542 | LPS |
| *S. paucissectum* | *S. paucissectum* | 202 | 29 | 0.990099 | 10 | 10 | 6.9967 | LPS |
| *S. peloquinianum* |  | 10 | 8 | 2 | 4.220245 | 5 | 0 | HPS |
| *S. pillahuatense* | *S. pillahuatense* | 16 | 12 | 0.625 | 10 | 10 | 0 | HPS |
| *S. pinnatisectum* |  | 4 | 3 | 7.5 | 10 | 10 | 0 | HPS |
| *S. piurae* | *S. piurae* | 243 | 43 | 0.699588 | 0.467511 | 2.727273 | 1.298124 | HPS |
| *S. polyadenium* | *S. polyadenium* | 385 | 102 | 2.571429 | 3.521418 | 7.647059 | 4.579969 | MPS |
| *S. puchupuchense* | *S. candolleanum* | 4 | 3 | 0 | 0 | 0 | 0 | HPS |
| *S. raphanifolium* | *S. raphanifolium* | 817 | 260 | 2.692778 | 6.517477 | 7.5 | 5.570085 | LPS |
| *S. raquialatum* |  | 18 | 11 | 1.666667 | 10 | 5 | 0 | HPS |
| *S. regularifolium* | *S. andreanum* | 3 | 3 | 6.666667 | 10 | 10 | 0 | HPS |
| *S. rhomboideilanceolatum* | *S. rhomboideilanceolatum* | 13 | 7 | 0.769231 | 10 | 10 | 0 | HPS |
| *S. salasianum* | *S. salasianum* | 13 | 7 | 0 | 0 | 0 | 0 | HPS |
| *S. sanctae-rosae* | *S. boliviense* | 2 | 1 | 5 | NA | NA | 0 | HPS |
| *S. sandemanii* | *S. medians* | 15 | 7 | 0.666667 | 10 | 6.666667 | 0 | HPS |
| *S. santolallae* | *S. laxissimum* | 11 | 10 | 2.727273 | 0.291422 | 2.307692 | 0 | HPS |
| *S. sarasarae* | *S. candolleanum* | 10 | 6 | 5 | 10 | 7.5 | 0 | HPS |
| *S. saxatilis* | *S. candolleanum* | 5 | 4 | 6 | 0.614619 | 2.857143 | 0 | HPS |
| *S. scabrifolium* |  | 6 | 5 | 3.333333 | 8.143671 | 4 | 0 | HPS |
| *S. schenckii* | *S. schenckii* | 154 | 42 | 3.181818 | 2.446487 | 6.538462 | 4.055589 | MPS |
| *S. simplicissimum* |  | 8 | 8 | 1.25 | 10 | 10 | 0 | HPS |
| *S. soestii* |  | 4 | 1 | 0 | NA | NA | 0 | HPS |
| *S. sogarandinum* | *S. sogarandinum* | 184 | 91 | 1.467391 | 3.222323 | 6 | 3.563238 | MPS |
| *S. solisii* | *S. andreanum* | 2 | 2 | 10 | 10 | 10 | 0 | HPS |
| *S. sparsipilum* | *S. brevicaule* | 125 | 78 | 4.72 | 2.931693 | 5 | 4.217231 | MPS |
| *S. spegazzinii* | *S. brevicaule* | 11 | 3 | 8.181818 | NA | 10 | 0 | HPS |
| *S. stenophyllidium* |  | 24 | 11 | 9.583333 | 10 | 10 | 9.861111 | NFCR |
| *S. stipuloideum* |  |  |  |  |  |  |  | HPS |
| *S. stoloniferum* | *S. stoloniferum* | 5389 | 1665 | 2.93561 | 10 | 10 | 7.645203 | NFCR |
| *S. subpanduratum* | *S. colombianum* | 1 | 1 | 0 | NA | NA | 0 | HPS |
| *S. sucubunense* | *S. colombianum* | 1 | 1 | 10 | NA | NA | 0 | HPS |
| *S. tacnaense* | *S. medians* | 24 | 12 | 2.916667 | 7.004689 | 10 | 0 | HPS |
| *S. tapojense* | *S. candolleanum* | 6 | 4 | 3.333333 | 7.219183 | 4 | 0 | HPS |
| *S. tarapatanum* | *S. candolleanum* | 9 | 6 | 4.444444 | 10 | 10 | 0 | HPS |
| *S. tarijense* | *S. berthaultii* | 32 | 19 | 8.4375 | 7.122184 | 10 | 8.519895 | NFCR |
| *S. tarnii* | *S. tarnii* | 113 | 37 | 3.982301 | 2.580262 | 4.285714 | 3.616092 | MPS |
| *S. taulisense* | *S. chomatophilum* | 2 | 2 | 0 | NA | NA | 0 | HPS |
| *S. trifidum* |  | 4 | 2 | 10 | 10 | 10 | 0 | HPS |
| *S. trinitense* |  | 8 | 7 | 2.5 | 4.770455 | 5 | 0 | HPS |
| *S. tundalomense* | *S. colombianum* | 20 | 8 | 10 | 4.880607 | 5 | 6.626869 | LPS |
| *S. tuquerrense* | *S. andreanum* | 25 | 20 | 5.2 | 10 | 10 | 8.4 | NFCR |
| *S. ugentii* | *S. brevicaule* | 33 | 13 | 6.060606 | 1.128508 | 4.285714 | 3.824943 | MPS |
| *S. urubambae* | *S. violaceimarmoratum* | 70 | 35 | 2.142857 | 0.945312 | 3 | 2.02939 | HPS |
| *S. velardei* | *S. candolleanum* | 21 | 15 | 3.333333 | 10 | 10 | 0 | HPS |
| *S. venturii* | *S. venturii* | 204 | 67 | 1.911765 | 0.471703 | 4 | 2.127823 | HPS |
| *S. vernei* | *S. vernei* | 690 | 161 | 3.782609 | 2.45927 | 8.181818 | 4.807899 | MPS |
| *S. verrucosum* | *S. verrucosum* | 1190 | 394 | 1.865546 | 6.562243 | 5.652174 | 4.693321 | MPS |
| *S. vidaurrei* | *S. brevicaule* | 14 | 10 | 10 | 1.123741 | 5 | 5.37458 | LPS |
| *S. vilgultorum* | *S. brevicaule* | 1 | 1 | 10 | 10 | 10 | 0 | HPS |
| *S. violaceimarmoratum* | *S. violaceimarmoratum* | 16 | 8 | 7.5 | 0.95694 | 3.076923 | 3.844621 | MPS |
| *S. wittmackii* |  | 60 | 37 | 3.333333 | 1.668464 | 2.5 | 2.500599 | HPS |
| *S. woodsonii* | *S. colombianum* | 50 | 3 | 0.6 | NA | 10 | 0 | HPS |
| *S. yamobambense* |  | 2 | 2 | 0 | NA | NA | 0 | HPS |
| *S. yungasense* | *S. chacoense* | 12 | 11 | 3.333333 | 0.235697 | 2.352941 | 0 | HPS |
