# Supplementary material for: Ex Situ Conservation Priorities for the Wild Relatives of Potato (Solanum L. Section Petota)
Source: PLoS One. 2015 Apr 29;10(4):e0122599. doi: 10.1371/journal.pone.0122599 (PMC4414521; doi:10.1371/journal.pone.0122599)
Supplement: S2 Table — C.V.: coefficient of variation (DOCX) [file pone.0122599.s006.docx]

**S2 Table. List of bioclimatic variables [99] used as environmental drivers to produce environmental niche models.** C.V.: coefficient of variation

| **Code** | **Description** | **Units** |
| --- | --- | --- |
| BIO 1 | Annual mean temperature | °C |
| BIO 2 | Mean diurnal range | °C |
| BIO 3 | Isothermality | % |
| BIO 4 | Temperature seasonality | % |
| BIO 5 | Max temperature of warmest month | °C |
| BIO 6 | Min temperature of coldest month | °C |
| BIO 7 | Temperature annual range | °C |
| BIO 8 | Mean temperature of wettest quarter | °C |
| BIO 9 | Mean temperature of driest quarter | °C |
| BIO 10 | Mean temperature of warmest quarter | °C |
| BIO 11 | Mean temperature of coldest quarter | °C |
| BIO 12 | Annual precipitation | mm |
| BIO 13 | Precipitation of wettest month | mm |
| BIO 14 | Precipitation of driest month | mm |
| BIO 15 | Precipitation seasonality | C.V. |
| BIO 16 | Precipitation of wettest quarter | mm |
| BIO 17 | Precipitation of driest quarter | mm |
| BIO 18 | Precipitation of warmest quarter | mm |
| BIO 19 | Precipitation of coldest quarter | mm |
