# Supplementary material for: Ex Situ Conservation Priorities for the Wild Relatives of Potato (Solanum L. Section Petota)
Source: PLoS One. 2015 Apr 29;10(4):e0122599. doi: 10.1371/journal.pone.0122599 (PMC4414521; doi:10.1371/journal.pone.0122599)
Supplement: S3 Table — (DOCX) [file pone.0122599.s007.docx]

**S3 Table. High priority species for further collecting and the main factors contributing to insufficient representation in germplasm collections.**

| **Species** | **Sample Size (SZ) in genebanks (< 10 active accessions)** | **Sampling Representativeness Score (SRS)** | **Ecosystem Representativeness Score (ERS)** | **Geographical Representativeness Score (GRS)** |
| --- | --- | --- | --- | --- |
| *S. acroglossum* | X | X | X | X |
| *S. acroscopicum* |  | X |  | X |
| *S. ayacuchense* | X | X | X | X |
| *S. bombycinum* | X | X |  | X |
| *S. buesii* | X | X | X | X |
| *S. burkartii* | X | X |  |  |
| *S. cajamarquense* |  | X |  | X |
| *S. cantense* | X | X |  | X |
| *S. chilliasense* | X | X |  |  |
| *S. clarum* | X | X | X |  |
| *S. contumazaense* | X | X |  |  |
| *S. garcia-barrigae* | X | X | X | X |
| *S. gracilifrons* | X | X |  | X |
| *S. hastiforme* | X | X |  | X |
| *S. hintonii* | X | X | X | X |
| *S. hjertingii* |  | X |  | X |
| *S. hougasii* |  | X |  | X |
| *S. incasicum* | X | X |  |  |
| *S. laxissimum* |  | X |  | X |
| *S. limbaniense* |  | X |  | X |
| *S. lobbianum* | X |  |  |  |
| *S. maglia* |  | X | X | X |
| *S. neocardenasii* |  |  |  | X |
| *S. neovavilovii* | X | X | X | X |
| *S. nubicola* | X | X |  | X |
| *S. olmosense* | X | X | X | X |
| *S. pillahuatense* | X | X |  |  |
| *S. piurae* |  | X | X | X |
| *S. rhomboideilanceolatum* | X | X |  | X |
| *S. salasianum* | X | X | X | X |
| *S. venturii* |  | X |  | X |
| *S. violaceimarmoratum* |  | X | X | X |
