# Supplementary material for: Ex Situ Conservation Priorities for the Wild Relatives of Potato (Solanum L. Section Petota)
Source: PLoS One. 2015 Apr 29;10(4):e0122599. doi: 10.1371/journal.pone.0122599 (PMC4414521; doi:10.1371/journal.pone.0122599)
Supplement: S4 Table — (DOCX) [file pone.0122599.s008.docx]

**S4 Table. List of regions and localities where further collecting may be targeted per species.**

| **Taxon** | **Country** | **First administrative level** | **Second administrative level** |
| --- | --- | --- | --- |
| *S. acroglossum* | Peru | Pasco | Pasco, Daniel Alcides Carrión |
|  |  | Huánuco | Dos de Mayo, Huánuco, Lauricocha, Pachitea, Huamalíes |
| *S. acroscopicum* | Peru | Arequipa | Condesuyos, La Unión |
|  |  | Ayacucho | Lucanas, Parinacochas, Paucar del Sara Sara |
|  |  | Cajamarca | Cutervo |
|  |  | Moquegua | Mariscal Nieto, General Sánchez Cerro |
|  |  | Tacna | Tacna, Tarata |
| *S. ayacuchense* | Peru | Ayacucho | Huanta, La Mar |
| *S. bombycinum* | Bolivia | La Paz | Franz Tamayo, Sud Yungas |
| *S. buesii* | Peru | Cusco | Cusco, Urubamba, La Convención |
| *S. burkartii* | Peru | Amazonas | Luya |
|  |  | Cajamarca | Chota, Cutervo |
| *S. cajamarquense* | Peru | Cajamarca | Cajabamba, Cajamarca, Chota, San Marcos, San Miguel, San Pablo, Santa Cruz, Contumazá |
|  |  | Lambayeque | Chiclayo |
| *S. cantense* | Peru | Ancash | Aija, Casma, Huarmey, Pallasca, Sihuas |
|  |  | Lima | Cajatambo, Canta, Huaral, Huarochiri, Lima, Oyon, Yauyos |
| *S. chilliasense* | Ecuador | El Oro | Atahualpa, Chilla, Santa Rosa |
|  |  | Loja | Chaguarpamba |
| *S. clarum* | Guatemala | Alta Verapaz | San Pedro Carchá |
|  |  | Chimaltenango | San José Poaquil |
|  |  | Huehuetenango | Chiantla, San Sebastián Huehuetenango, San Sebastián Coatán, Todos Santos Cuchumatán |
|  |  | Quezaltenango | Coatepeque, Palestina de Los Altos, Quetzaltenango, San Carlos Sija, Sibilia |
|  |  | San Marcos | Ixchiguan, Tacaná |
|  |  | Sacatepéquez | Alotenango, Santa María de Jesús |
|  |  | Totonicapán | Momostenango, San Francisco El Alto, Totonicapán, Santa María Chiquimula, San Cristóbal Totonicapán |
|  |  | Sololá | Nahualá, Sololá |
|  | Mexico | Chiapas | El Porvenir, Motozintla |
| *S. contumazaense* | Peru | Cajamarca | Contumazá, Gran Chimú |
|  |  | La Libertad |  |
| *S. garcia-barrigae* | Colombia | Magdalena | Aracataca, Santa Marta (Dist. Esp.), Ciénaga |
|  |  | Norte de Santander | Abrego, Sardinata |
| *S. gracilifrons* | Peru | Huancavelica | Churcampa, Tayacaja |
| *S. hastiforme* | Peru | Amazonas | Chachapoyas |
|  |  | Ancash | Carhuaz, Corongo, Huaylas, Pallasca |
|  |  | La Libertad | Otuzco, Pataz, Santiago de Chuco, Sánchez Carrión |
| *S. hintonii* | Mexico | Colima | Cuauhtémoc |
|  |  | Guanajuato | Xichú |
|  |  | Mexico | Atlacomulco, Temascaltepec, Tlatlaya, Valle de Bravo, Zacazonapan |
|  |  | Queretaro | Cadereyta de Montes |
|  |  | San Luis Potosi | Villa de Ramos |
| *S. hjertingii* | Mexico | Aguascalientes | Calvillo |
|  |  | Baja California | Mexicali |
|  |  | Coahuila | Arteaga, Múzquiz, Parras, Ramos Arizpe, Saltillo, Acuña, Cuatrociénegas |
|  |  | Nuevo León | Aramberri, Galeana, Rayones, Santa Catarina |
|  |  | Puebla | Zacatlán |
|  |  | San Luis Potosi | Catorce, San Luis Potosí |
|  |  | Tamaulipas | Jaumave, Miquihuana |
| *S. hougasii* | Mexico | Chihuahua | Chihuahua, Gran Morelos |
|  |  | Colima | Colima, Cuauhtémoc |
|  |  | Guerrero | Atoyac de Alvarez, General Heliodoro Castillo |
|  |  | Jalisco | Autlán de Navarro, Ciudad Venustiano Carranza, Sayula, Tapalpa, Tecalitlán, Tonila, Tuxpan, Zapotitlán de Vadillo |
|  |  | Mexico | Villa Victoria |
|  |  | Michoacán | Cherán, Nuevo Paranguricutiro, Nuevo Urecho, Paracho, Pátzcuaro, Peribán, Salvador Escalante, Tacámbaro, Uruapan, Queréndaro |
| *S. incasicum* | Peru | Cajamarca | Cutervo |
|  |  | Cusco | Urubamba, La Convención |
| *S. laxissimum* | Peru | Ayacucho | Huanca Sancos, Huanta, La Mar |
|  |  | Cusco | Canas, Canchis, Chumbivilcas, Espinar, Paucartambo, Urubamba, La Convención |
|  |  | Pasco | Oxapampa |
|  |  | Huánuco | Huenuco, Leoncio Prado, Huamalíes |
|  |  | Junín | Chanchamayo, Huancayo, Jauja, Satipo, Tarma |
| *S. limbaniense* | Peru | Puno | Carabaya, Melgar, San Antonio de Putina, Sandia, Chucuíto |
| *S. maglia* | Argentina | Jujuy | Tilcara, Tumbaya |
|  |  | Mendoza | San Carlos |
|  | Chile | Coquimbo | Choapa, Elqui, Limarí |
|  |  | Maule | Linares, Talca |
|  |  | Valparaíso | Petorca, Valparaíso |
|  |  | Bío-Bío | Biobío |
| *S. neocardenasii* | Bolivia | Cochabamba | Chapare, Esteban Arce, Quillacollo |
|  |  | Santa Cruz | Florida, Manuel María Caballero, Vallegrande, Ñuflo de Chávez, José Miguel de Velasco, Andrés Ibáñez |
| *S. neovavilovii* | Bolivia | La Paz | Franz Tamayo |
| *S. nubicola* | Peru | La Libertad | Pataz |
|  |  | Lima | Lima |
|  |  | Piura | Huancabamba |
|  |  | Huánuco | Ambo, Huamalíes |
|  |  | Apurímac | Abancay |
| *S. olmosense* | Ecuador | El Oro | Santa Rosa, Piñas |
|  |  | Loja | Celica |
|  | Peru | Lambayeque | Lambayeque |
|  |  | Piura | Huancabamba, Sullana |
| *S. pillahuatense* | Peru | Cusco | Paucartambo, Urubamba |
|  |  | Apurímac | Abancay |
| *S. piurae* | Peru | Cajamarca | Cajamarca |
|  |  | La Libertad | Otuzco |
|  |  | Piura | Ayabaca, Huancabamba, Morropón |
| *S. rhomboideilanceolatum* | Peru | Huancavelica | Tayacaja |
|  |  | Junín | Huancayo |
| *S. salasianum* | Peru | Huánuco | Huánuco, Pachitea |
| *S. venturii* | Argentina | Catamarca | Ambato, Belén, Santa María, Andalgalá |
|  |  | Jujuy | Capital, Tilcara, Valle Grande |
|  |  | La Rioja | Vinchina |
|  |  | Salta | Cachi, Capital (Salta), Chicoana, Guachipas, Rosario de Lerma, Santa Victoria, Orán |
|  |  | Tucumán | Chicligasta, Simoca, Trancas, Tafí del Valle, Tafí Viejo |
| *S. violaceimarmoratum* | Bolivia | Cochabamba | Arani, Ayopaya, Carrasco, Cercado, Chapare |
|  |  | El Beni | José Ballivián |
|  |  | La Paz | Bautista Saavedra, Franz Tamayo, Inquisivi, Larecaja, Nor Yungas, Pedro Domingo Murillo, Sud Yungas |
|  | Peru | Cusco | Calca, Cusco, Paucartambo, Urubamba, La Convención |
